# Continuous injection synthesis of indium arsenide quantum dots emissive in the short-wavelength infrared

**DOI:** 10.1038/ncomms12749

**Published:** 2016-11-11

**Authors:** Daniel Franke, Daniel K. Harris, Ou Chen, Oliver T. Bruns, Jessica A. Carr, Mark W. B. Wilson, Moungi G. Bawendi

**Affiliations:** 1 Department of Chemistry, Massachusetts Institute of Technology, 77 Massachusetts Avenue, Cambridge, Massachusetts 02139, USA

## Abstract

With the emergence of applications based on short-wavelength infrared light, indium arsenide quantum dots are promising candidates to address existing shortcomings of other infrared-emissive nanomaterials. However, III–V quantum dots have historically struggled to match the high-quality optical properties of II–VI quantum dots. Here we present an extensive investigation of the kinetics that govern indium arsenide nanocrystal growth. Based on these insights, we design a synthesis of large indium arsenide quantum dots with narrow emission linewidths. We further synthesize indium arsenide-based core-shell-shell nanocrystals with quantum yields up to 82% and improved photo- and long-term storage stability. We then demonstrate non-invasive through-skull fluorescence imaging of the brain vasculature of murine models, and show that our probes exhibit 2–3 orders of magnitude higher quantum yields than commonly employed infrared emitters across the entire infrared camera sensitivity range. We anticipate that these probes will not only enable new biomedical imaging applications, but also improved infrared nanocrystal-LEDs and photon-upconversion technology.

## Abstract (web summary)

Indium arsenide quantum dots are promising materials for short-wavelength infrared emissive applications. Here, the authors investigate the kinetics of indium arsenide nanocrystal growth and design large quantum dots with narrow emission wavelengths which can be used for through-skull fluorescence imaging.

## Graphical abstract

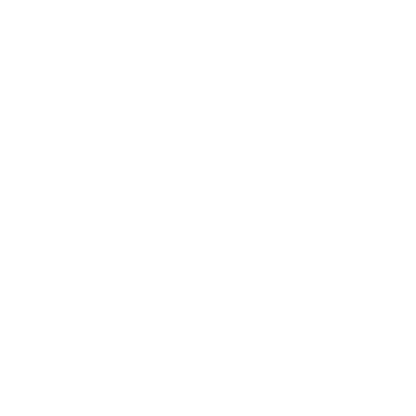

Technological improvements in the fabrication of short-wavelength infrared (SWIR, 1,000–2,000 nm) detector technology have recently inspired a new wave of optical fluorescence imaging, as longer imaging wavelengths promise increased spatiotemporal resolution, penetration depths and unprecedented sensitivity^1^,^2^,^3^,^4^. However, with the emergence of SWIR imaging, the shortage of high-quality SWIR-emissive fluorophores has never been more apparent: the ideal imaging probe needs to exhibit high brightness for fast imaging speeds and narrow emission profiles that can be tuned over the entire SWIR camera sensitivity range for multiplexed and wavelength-selective imaging. Furthermore, probes should exhibit high stability of the quantum yield (QY) and wavelength of the emission peak, both under laser irradiance as well as during long-term storage. Lastly, and with respect to potential probe commercialization, fluorophore synthesis should run reproducibly at high yields.

Indium arsenide (InAs) quantum dots (QDs) are among the most promising SWIR probes to address these challenges^5^,^6^, as they exhibit size-tunable emission, broad absorption spectra, and show higher QYs than rare earth nanocrystals (NCs)^7^,^8^, silver chalcogenide NCs^9^, or organic SWIR dyes^10^. While much recent SWIR imaging has focused on carbon nanotubes (CNTs)^2^,^11^,^12^, the low QYs (<0.1%) and broad emission profiles of as-synthesized CNT ensembles have rendered imaging in narrow spectral windows and multiplexed imaging applications challenging. In contrast to other SWIR QDs, such as PbS or Ag_2_S, InAs QDs can exhibit higher QYs and probe stability after transfer from the organic phase to aqueous media^5^,^13^. This is mostly attributed to the zincblende crystal structure of InAs QDs that allows the straightforward overcoating with a higher band gap shell consisting of established II–VI QD materials, which isolates the InAs core from the environment^5^,^14^. However, the synthesis of large, SWIR-emissive, InAs NCs with narrow emission linewidths remains challenging and conventional syntheses require additional post-synthetic purification, which drastically reduces reaction yields. Thus, to obtain fluorescent emitters in the red edge of the sensitivity range of SWIR cameras, small, near-infrared-emissive InAs QDs have been commonly overcoated with other materials to form quasi type-II heterostructures, which further redshift the emission. InAs-based QDs emitting beyond 1,400 nm have previously been characterized by low QYs (with the best reported value to our knowledge of 2.5%), and photobleaching of 50% after 2 h of irradiation^5^.

In this study, we present an improved understanding of the underlying mechanisms of InAs QD growth and describe a new synthetic route that overcomes the shortcomings of previous methods. Our approach enables the synthesis of InAs QDs with narrow emission (<135 meV linewidths) tunable from 700–1,200 nm without employing size-selective purification procedures. Based on these InAs QD cores, we develop improved core-shell-shell (CSS) QDs that span the entire sensitivity range of modern SWIR cameras (900–1,600 nm, http://www.princetoninstruments.com/products/imcam/nirvana/), exhibit QYs up to 82%, and show drastically improved probe stability both under laser irradiance and during long-term storage. With QYs of 16% at the red edge of the sensitivity range of SWIR cameras, our probes are more than six times brighter than previously published InAs-based QD systems and further maintain high QYs in aqueous media after phase transfer. Ultimately, we demonstrate how our SWIR fluorophores exhibit a 2–3 order of magnitude higher QY than CNTs^2^,^12^ across the entire sensitivity range of SWIR cameras and demonstrate their use in non-invasive through-skull fluorescence imaging in mice. The improved material properties position our InAs CSS QDs as the material of choice for SWIR fluorescence imaging and suggest possible use in other applications such as SWIR LEDs, photovoltaics, photodetectors and photon upconversion devices^15^,^16^,^17^,^18^.

## Results

### Current challenges in the synthesis of InAs QDs

Most synthetic approaches towards the growth of InAs QDs can be divided into two categories, based on the choice of the indium precursor. On one hand, the use of indium halides^19^,^20^ typically yields NCs with sizes ranging from 2–6 nm, though syntheses require size-selective purification to obtain narrow size distributions with well-defined absorption features, which is costly and impractical to scale up. In contrast, syntheses that use indium carboxylates are able to obtain narrow size distributions without requiring size-selective precipitation^21^. However, these syntheses rely on an interparticle ripening process as a primary growth mechanism. Thus, the obtainable NC size range remains limited to small sizes and denies access to QDs emitting in the sensitivity range of SWIR cameras.

To address the existing challenges in the synthesis of InAs QDs, it is crucial to understand the mechanisms that govern particle formation and growth. Predictions from theoretical growth models^22^,^23^,^24^ and experimental results^22^,^25^,^26^ for II–VI and IV–VI QD systems suggest that tight control of precursor conversion rates will result in the ability to tune size and size-distribution of the resulting QD samples. Recent results of Hendricks *et al*.^27^ have elegantly shown how chalcogenide precursors with different reactivities can be used to synthesize a broad size range of cadmium sulfide and lead sulfide QDs with narrow emission profiles at full reaction yields. While for those systems, precursor conversion and particle growth occur on similar timescales, we have previously shown that for common III–V QD syntheses the group-V precursors employed are consumed on a much faster timescale than the actual NC growth, implying that QD growth is dominated by non-molecular ripening processes^28^. Based on this observation, it was widely believed that less-reactive group-V precursors would be able to improve the control of III–V QD syntheses, which inspired multiple groups to examine group-V precursor reactivity^29^,^30^,^31^. However, it has recently been shown that changes in precursor reactivity play an unexpectedly minor role in the growth of III–V QDs and do not enable the synthesis of large InAs crystals^29^,^32^.

### The role of precursor conversion rates

Precursor conversion rate (Eq. 1) is a function of the precursor reactivity, manifested in the precursor conversion rate constant *k*, as well as the time-dependent concentration of precursor molecules in the reaction mixture.


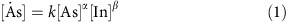


**Figure 1:**
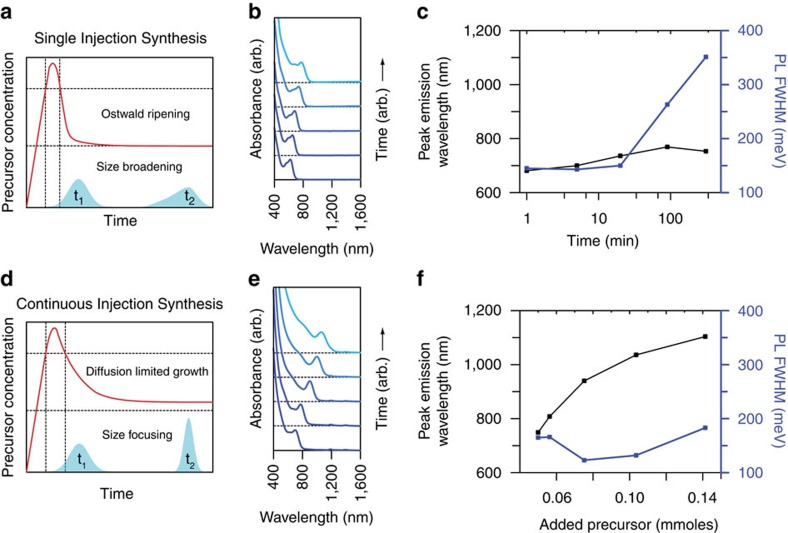
Growth mechanisms for InAs QDs. Schematic representation of the temporal evolution of precursor concentration during (**a**) a typical hot-injection synthesis of InAs QDs and (**d**) during a combination of hot and continuous injection. As a single injection supplies all precursor material at the beginning of the reaction, growth is primarily dominated by interparticle ripening, leading to a small overall growth of nanocrystals that is correlated with a broadening of the size distribution at long time scales (**b**,**c**). If a single injection, however, is followed by a slow, continuous supply of precursor material, the system can be maintained in a prolonged size-focusing regime, giving access to large nanocrystals with narrow size distributions (**e**,**f**).

Following this rationale, we investigated to what extent control of the time-dependent precursor concentration could improve the synthesis of InAs QDs. Figure 1a–c shows a typical hot-injection synthesis of InAs QDs, using an excess of indium carboxylates and tris(trimethylgermyl)arsine, (TMGe)_3_As, as precursors^30^. Due to the high reactivity of (TMGe)_3_As, both precursors are rapidly consumed upon mixing and, following nucleation, the size of the QDs barely changes over the remaining course of the reaction, as indicated by the minimal (<100 nm) change in the peak wavelength of the emission (Fig. 1c). Additionally, if the reaction is left in this state for an extended period of time, we observe a strong broadening of the size distribution, here reflected by the full width at half maximum (FWHM) of the photoluminescence (PL) peak. This is evidence for the onset of Ostwald ripening, which increases the mean particle size through interparticle interactions^33^.

In our new approach, we only employ 10% of the total amount of arsenic precursor used in Fig. 1a–c to induce a nucleation event and supply the residual amount slowly via a syringe pump. As the time scale of precursor reactivity is much faster than the precursor addition rate, the precursor conversion rate and thus QD growth is ultimately limited by the precursor supply. Consequently, this method allows the precursor conversion kinetics to be independently tuned in the nucleation and growth stages by precisely adjusting the time-dependent precursor concentration profile via syringe pump. Figure 1d,e and Supplementary Fig. 1 show how such an approach allows the continuous growth of NCs with tunable emission from 700–1,200 nm while maintaining narrow size distributions. Further, we note that the decoupling of the precursor reactivity and growth kinetics offered by our method enables the growth of QDs from reactive precursors at high reaction temperatures (295 °C), which improve crystal quality^34^.

### Kinetic insights into III–V QD growth

**Figure 2:**
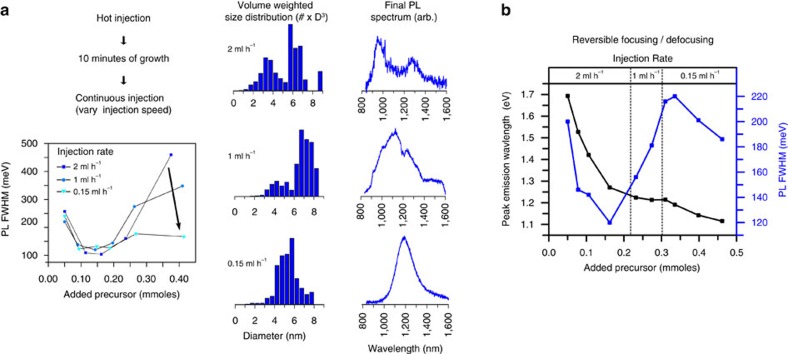
Kinetic insights into the growth of InAs QDs. (**a**) Influence of the injection rate on the extent of size-focusing. At early stages of QD growth, size-focusing appears invariant to injection speed. However, at later stages, QD growth slows down and fast injection speeds lead to a secondary nucleation event, resulting in a bimodal size distribution, as identified by TEM and two distinct PL peaks (the feature at 1,200 nm in the emission spectrum of the 1 ml h^−1^ injection rate originates from solvent reabsorption). Slow injection speeds, however, are able to maintain the system in a size-focused regime, providing access to large particles with narrow size distributions. (**b**) Variation of injection speed during growth within a single synthesis shows the capabilities of our approach. At the beginning of the reaction fast injection speeds bring the system into a size-focusing regime. As soon as growth slows down, as can be seen from the PL peak values slowly converging over time, size-broadening sets in. A drastic decrease in injection rate, however, can be used to push the system back into a size-focusing growth regime, yielding a second onset of QD growth and a partial reversion of the occurred size-broadening.

To probe the extent to which precursor addition rate affects QD growth, we varied the injection speed of the arsenic precursor ((TMGe)_3_As) from 0.15 ml h^−1^ to 1 ml h^−1^ to 2 ml h^−1^. Figure 2a shows that for all three injection speeds an initial size-focusing is observed. However, for the fastest addition rate the QD size distribution significantly broadens after about 50% of the reaction time, yielding a bimodal final size distribution evident from transmission electron microscopy (TEM) and two distinct PL peaks. We observed a similar trend in size broadening for the intermediate injection speed, which we found to be caused by shape anisotropy (see Supplementary Note 1, Supplementary Figs 2 and 3). By contrast, synthesis at the slowest injection speed yields a narrow emission peak at about 1,200 nm and a unimodal size distribution.

To explain the reduced synthetic quality at elevated injection rates, we propose that the higher concentration of precursor material in solution leads to continuous and/or secondary nucleation events. Thus, whereas previous studies on InAs and CdSe QD growth concluded that size defocusing primarily occurs due to the absence of precursor material in solution^33^,^35^, our study shows that elevated precursor concentrations can likewise cause size-broadening. Accordingly, we consider that there is an optimal precursor conversion rate for the synthesis of high-quality QDs where the growth remains size-focused—simultaneously avoiding ripening processes that occur at either extreme of precursor injections speeds.

However, the injection rate-dependent size focusing that we observe implies that the optimal precursor conversion rate varies over the course of the synthesis. Specifically, given that the injection rate is constant during each of the three experiments shown in Fig. 2a, the varying extent of size focusing at different injection rates implies that the precursor demand by the growing NCs changes over the course of the synthesis. Because we obtain improved results at the slowest injection rate, we consider that the size-broadening at late times results from an excess of converted precursor in the growth solution, which indicates that NC growth slows down as NC size increases.

To test this hypothesis, we demonstrate that the growth of InAs QDs can be reversibly switched between a size-focusing and a size-broadening state via gradual adjustment of the injection speed from fast to slow (Fig. 2b). As seen in Fig. 2a, size-focusing occurs at the beginning of the reaction even at elevated injection speeds as the growth of small particles is fast enough to accommodate for high precursor concentrations. As crystals reach a size of ∼4.5 nm, QD growth slows down, and the PL peak saturates around 1.2 eV. Meanwhile, the PL FWHM drastically increases from 120 to 220 meV. Upon slowing down precursor injection speed to 0.15 ml h^−1^, we note a second onset of NC growth, indicated by a redshift of the emission peak. Importantly, this NC growth is accompanied by a narrowing of the PL FWHM, showing that we have switched the system from a size-broadening state back into a size-focusing growth regime.

Together our results reveal that the ideal growth conditions for InAs QDs significantly differ for smaller clusters and larger particles. Neither typical hot injection approaches that focus on the nucleation event and the growth of smaller particles^21^, nor syntheses that employ multiple precursor injections^35^,^36^ maintain the QD system in a size-focusing growth regime at larger crystals sizes due to elevated precursor concentrations. Our continuous injection synthesis allows us to adjust the precursor conversion kinetics for both growth phases separately and precisely, giving access to large crystals with narrow size distributions. Based on these observations, we were able to significantly decrease overall reaction times by running the synthesis of large crystals at high initial precursor addition rates, followed by slower addition rates once QDs reached a size of about 4 nm and crystal growth significantly slowed down (see Supplementary Figs 4 and 5). As controlling precursor conversion kinetics via syringe pump has recently also allowed the synthesis of novel QD materials such as Cd_3_As_2_ (ref. 37), our results highlight the broad applicability of the continuous injection approach for the synthesis of challenging nanoparticle systems. In Supplementary Note 2 and Supplementary Fig. 6 for example, we show that a similar synthetic approach can be used to grow larger InP QDs with narrow emission linewidths from small seeds.

### Continuous injection overcomes precursor identity

As the reaction kinetics of our approach are governed by the precursor addition rate, the influence of the molecular identity of the arsenic precursor should be minimal. Specifically, if two different precursors both react on a faster time scale than the precursor addition rate, then the growth of InAs QDs from either precursor should be similar. To verify this hypothesis we compared the results of our (TMGe)_3_As-based synthesis (Fig. 1d,e) with syntheses employing either a more reactive precursor (tris(trimethylsilyl)arsine) or a less reactive precursor (tris-(isopropyldimethylsilyl)arsine) (see Supplementary Figs 4 and 5; ref. 32).We obtained InAs QDs with comparable size and electronic polydispersity by all three routes, confirming our understanding. Moreover, we emphasize that the use of less reactive arsenic precursors in our continuous injection approach is a major safety improvement and paves the way for a broader application of InAs QDs—especially if our results can be extended to arsenic precursors that are safer to work with and to synthesize.

### Stable SWIR-emitting CSS QD

As-synthesized InAs QDs for applications in light emission are commonly overcoated with higher band gap semiconducting materials, such as CdSe, CdS, ZnS or ZnSe^5^,^6^,^19^. The epitaxial growth of a higher band gap semiconducting material passivates surface atoms, while confining the exciton within the core and protecting it from environmental influences^38^,^39^. Aharoni *et al*.^5^ have developed InAsCdSeZnSe CSS QDs, in which both shells serve distinct purposes. The first shell (CdSe) is lattice-matched to the InAs core, and yields a strong redshift of the PL peak with increasing shell thickness. This is indicative of a quasi type-II structure, likely due to the small conduction band offset between InAs and CdSe^14^,^40^. The outer shell (ZnSe) is thought to form a type-I heterojunction, confining the exciton to the core-shell region^5^. However, as the InAs cores employed previously were quite small, a very large CdSe shell was needed to shift the emission past 1,300 nm. The resulting CSS QDs exhibited low QYs at long emission wavelengths (2.5% at 1,425 nm) and showed significant photobleaching—such that the PL intensity of a sample in solution decreased to half its original intensity after illumination with a 30 mW laser at 437 nm (ref. 5). This highlighted an inherent trade-off of extended quasi type-II structures, as the increased electron delocalization tends to lengthen radiative lifetimes and strengthen exciton-phonon coupling—doubly reducing QY^14^,^41^,^42^.

**Figure 3:**
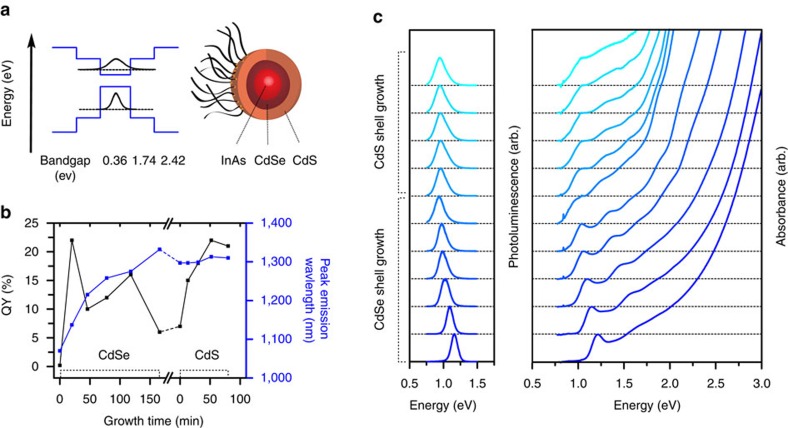
Synthesis of core-shell-shell QDs. (**a**) Schematic band alignment in InAsCdSeCdS CSS QDs, based on values provided in refs 14, 43. (**b**) Growth of two shells onto an InAs QD. While the CdSe shell leads to a strong redshift in the PL peak, the QY initially increases and then decreases during shell growth, indicative of the formation of a quasi type-II structure. The position of the PL peak stays approximately constant during growth of the outer CdS shell, while the QY shows steady improvements, indicative of the formation of a type-I structure. (**c**) Respective PL and absorbance spectra for the growth of InAsCdSeCdS QDs.

**Figure 4:**
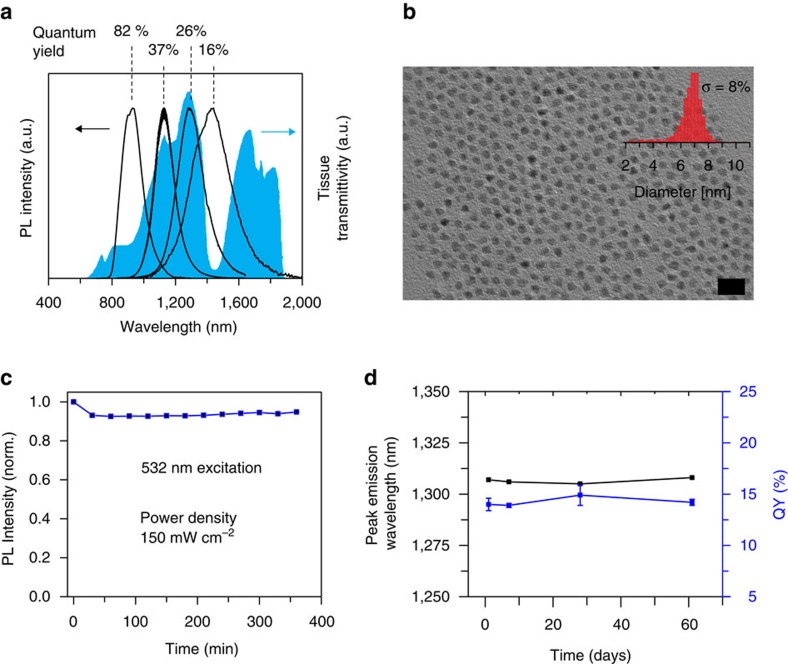
CSS SWIR QDs for wavelength-selective SWIR imaging. (**a**) CSS QDs spanning the entire sensitivity range of modern InGaAs cameras for wavelength-selective SWIR imaging. Fluorescence spectra are overlaid with a curve for the transmittivity of biological tissue, adapted from ref. 45. (**b**) Representative TEM image of InAsCdSeCdS CSS QDs with a size distribution of 8%. Scale bar, 20 nm. (**c**) PL Stability of CSS QD over time during illumination with a 532 nm diode laser, with an irradiance of 150 mW cm^−2^, which is in the range of the flux used in biomedical imaging applications^2^. (**d**) Long-term stability of dispersions of CSS QDs upon storage in open air. QY and position of PL maximum are well maintained for at least two months. Error bars (s.d.) originate from repeating each QY measurement three times.

Here we present new InAsCdSeCdS and InAsCdSeZnS CSS QDs (Fig. 3a) that employ InAs cores synthesized via our continuous injection approach. Large InAs cores enable the use of a thinner quasi type-II shell to redshift the emission into the deep SWIR, maintaining high QYs and probe stability at long emission wavelengths. Additionally, our InAs QDs show high-thermal stability and thus allow for shell growth at up to 280 °C in a 1:1 mixture of oleylamine and 1-octadecene (see Supplementary Fig. 7). Using cadmium oleate and a solution of selenium in trioctylphosphine for the growth of the inner shell, we obtained additional redshifts of more than >300 nm (see Supplementary Fig. 8). Figure 3b shows the correlation of QY and position of the PL peak during the growth of a CdSe shell onto an InAs core. While the QY initially rises drastically, likely caused by the passivation of surface atoms, it subsequently decreases with growing shell thickness, indicative of the formation of a quasi type-II structure and reduced electron-hole overlap^5^,^40^. To further confine the exciton within the InAsCdSe core-shell structure, we employed an additional outer high band gap shell, consisting of either CdS or ZnS. While the QY continuously increases during the growth of the outer shell, we note a small redshift of the emission peak for the growth of the CdS shell, likely due to a small offset of the conduction bands of CdSe and CdS^14^,^43^. For the growth of ZnS a small blueshift was detected. Both methods were able to produce samples with similarly high QYs (see Supplementary Fig. 9), such that the outer shell allows for a further tuning of the emission wavelength with high precision. In contrast to previous publications, the high-thermal stability of the InAs cores allowed us to employ non-pyrophoric compounds (cadmium oleate or zinc oleate and elemental sulfur) as respective outer shell precursors^5^,^6^. TEM images show narrow size distributions (Fig. 4b and Supplementary Fig. 10) and the presence of all elements was confirmed via energy dispersive X-Ray spectroscopy (Supplementary Tables 1 and 2).

In summary, the CSS structure allowed us to synthesize InAs-based CSS QDs samples spanning the entire sensitivity range of modern InGaAs cameras with QYs ranging from 82–16% (Fig. 4a). Our CSS QDs also show improved stability with regard to long-term storage and under high excitation flux. While previous InAs-based SWIR QDs lose roughly 50% of their initial fluorescence intensity due to photobleaching, our new generation of CSS QDs exhibits high photostability and shows <10% photobleaching upon irradiation with a 532 nm excitation source at flux intensities used in biomedical imaging (Fig. 4c; ref. 5). Dispersions of CSS QDs exhibit high long-term stability of the QY and the position of the PL peak in open air over at least two months (Fig. 4d). Even CSS QDs stored as a dried powder in air lose <5% of their initial QY after one month of storage. Further, in contrast to InAsCdSe core-shell QDs we synthesized, the PL lifetime traces of our CSS QDs exhibit an improved monoexponential character, indicating reduced non-radiative decay channels and enhanced sample homogeneity (Supplementary Fig. 11).

Lastly, our CSS QDs are readily transferred to aqueous media while maintaining colloidal stability and brightness by incorporating them into polyethylene glycol (PEG)-phospholipid micelles^44^. Using this straightforward phase transfer, we obtained water-soluble samples emitting at 970, 1,110, 1,300 and 1,430 nm with respective intrinsic QYs of 31, 23, 10 and 5%, corrected for reabsorption by water (see Supplementary Note 3 and Supplementary Fig. 11).

### SWIR bandpass filter imaging

**Figure 5:**
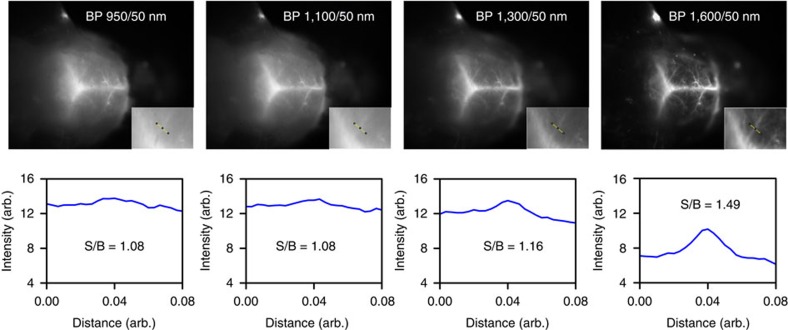
Wavelength-selective SWIR imaging with CSS QDs. Fluorescence imaging of mouse brain vasculature through intact skin and skull using a mixture of SWIR CSS QDs. The four images were collected using four bandpass filters (50 nm spectral width) centered at 950, 1,100, 1,300 and 1,600 nm with integration times of 150, 50, 100 and 5,000 ms, respectively. The images show that longer imaging wavelengths enhance the spatial resolution of fine vascular brain structures by significantly improving the signal to background (S/B) ratio in the region of interest, which stresses the need for SWIR emitters with high QYs and narrow emission at the red edge of SWIR detectors.

Ultimately, we employed CSS QDs to perform through-skull fluorescence imaging in mice^2^. As the optical properties of biological tissue are strongly wavelength-dependent within the sensitivity range of modern InGaAs-based SWIR cameras (900–1,600 nm), even slight changes in the imaging wavelength yield significantly different performance^1^,^45^. To demonstrate the effect of wavelength on SWIR imaging, we prepared a broadband SWIR emitter covering the entire sensitivity range of our InGaAs camera, by mixing three samples of CSS QDs emitting at 970, 1,110 and 1,300 nm. The solution was injected into an anaesthetized mouse (C57BL/6J) via the tail vein and the vasculature of the mouse brain was imaged through the intact skin and skull using diffuse 808 nm excitation. Figure 5 shows wavelength-selective images acquired at 950, 1,100, 1,300 and 1,600 nm using bandpass filters with a spectral width of 50 nm. It is evident that longer imaging wavelengths lead to enhanced resolution of the vascular structure of the mouse brain and improved image contrast. However, tissue transmittivity decreases beyond 1,300 nm (ref. 1), causing imaging speed to drastically reduce. The tradeoff between increased resolution and decreased imaging speed at longer imaging wavelength stresses the need for fluorescent emitters with high QYs and tunable emission linewidths that maximize the ratio of emitted photons per given spectral region. Our new generation of CSS QDs achieves a 2–3 order of magnitude increase in QY compared with commonly employed SWIR-emissive CNTs^2^,^12^ across the entire sensitivity range of modern SWIR cameras while exhibiting narrow emission linewidths. As a result, CSS QDs not only facilitate multiplexed fluorescence imaging, but also enhance imaging speed for high-resolution imaging at long imaging wavelengths through improved particle quality and the resulting enhancement in QY.

## Discussion

In conclusion, we demonstrate a new approach towards high-quality SWIR-emissive InAs QDs. Using a continuous injection approach, we studied the processes that govern III–V QD growth and revealed a significant decrease in the growth rate of larger InAs QDs. Adjusting the time-dependent precursor concentrations throughout QD growth allowed us to compensate for the shortcomings of previous syntheses and pave the way for the broader application of InAs QDs through the use of safer precursor materials. In contrast to the recent demonstration of Hendricks *et al*.^27^ that showed how a library of QD precursors with different reactivities can be used to synthesize broad size ranges of PbS and CdS QDs, our study suggests that a continuous injection approach can obtain identical results for a broad range of QD systems, simply by adjusting duration and speed of the precursor injection. Using large, high-quality InAs cores, we were able to synthesize CSS QDs with bright emission at wavelengths between 1,300–1,500 nm. These particles are more than six times brighter than previous InAs-based QDs (see Supplementary Fig. 12) and exhibit significantly improved stability. We further show that CSS QDs can be used to perform through-skull SWIR imaging in murine models at wavelengths spanning the entire sensitivity range of modern InGaAs cameras, including highly absorptive spectral regions.

## Methods

### Chemicals and reagents

Octadecene (90%), oleylamine (70%), oleic acid (90%), CdO, ZnO, selenium and sulfur were bought from Sigma-Aldrich and used as purchased. Trioctylphosphine (97%) was purchased from Strem Chemicals. Before use, all solvents were degassed and stored over molecular sieves if not used immediately. Indium(III) acetate (InAc_3_, 99.99%) was purchased from Alfa Aesar. Myristic acid (>98%) was purchased from Spectrum Chemical MFG Corp. Indium(III) myristate (InMy_3_), (TMSi)_3_As, (TMGe)_3_As and (iPrDMSi)_3_As were synthesized as described earlier^32^.

### Continuous injection synthesis

InAc_3_ (1 mmoles) and oleic acid (4 mmoles) were mixed in 1-octadecene (5 ml) in a four neck flask and degassed at room temperature for 30 min to yield a pressure <10 mtorr. The mixture was heated to 115 °C for another 60 min, during which a clear solution was formed. The atmosphere was switched to nitrogen and the temperature increased to 295 °C. In a glovebox (TMGe)_3_As (0.05 mmoles) was dissolved in TOP (1 ml) and subsequently injected into the solution to induce nucleation. After 10 min of reaction time (TMGe)_3_As (0.42 mmoles) in octadecene (2.5 ml) were slowly injected into the solution using a syringe pump and a cannula connecting syringe and flask through a septum. The injection speed was set to 0.15 ml h^−1^ and the reaction was run for another 900 min. The reaction was monitored by removing aliquots from the growth solution, diluting aliquots in organic solvents (hexanes or CCl_4_), and measuring the respective PL and absorption spectra. Subsequently, the heat was removed and the growth solution was transferred to a glovebox. QDs were precipitated from the growth solution using a mixture of 1-butanol and methanol and centrifuged for 3 min at 3,800 r.p.m. QDs were redispersed in hexanes and the process was repeated one more time.

### Shell precursors stock solutions

A 0.2 M solution of cadmium oleate (Cd(Ol)_2_) was obtained by mixing CdO (8 mmoles) with oleic acid (64 mmoles). The solution was degassed at room temperature for 1 h, and then degassed at 100 °C for another 2 h. The atmosphere was switched to nitrogen and heated to 200 °C until the solution turned clear. Subsequently, octadecene (20 ml) was added to the solution. A 0.1 M solution of zinc oleate (Zn(Ol)_2_) was obtained following the same procedure while substituting CdO for ZnO and adding additional 20 ml of oleylamine to the solution. A 0.2 M solution of TOPSe was obtained by mixing Se (4 mmoles), TOP (4 ml) and octadecene (16 ml) inside a glovebox and sonicating the resulting solution for 30 min. A 0.045 M solution of sulfur in octadecene was obtained by mixing sulfur (58 mg) in octadecene (40 ml) and heating the solution under vacuum at 110 °C for 2 h.

### CSS QD synthesis

For CSS QD synthesis InAs QDs were synthesized using (TMGe)_3_As or (TMSi)_3_As as a precursor and purified as described above. The diameter and the concentration of the synthesized particles in solution was determined by using a sizing curve^46^. Based on QD size a rough estimate of shell precursor material that is necessary to grow a certain amount of shell monolayers was calculated^5^. However, as InAs QDs do not exhibit a perfectly spherical shape the actual amount of deposited shell material per shell monolayer was found to slightly vary from the calculated values. The shell growth process was monitored by taking frequent aliquots throughout the growth process to determine absorption and fluorescence spectra, as well as QY values such that the final emission peak could be tuned to the desired wavelength. In a typical reaction, purified InAs QDs (96 nmoles, diameter of 4.7 nm, PL emission at 1,039 nm) in hexanes were transferred to a mixture of octadecene (3 ml) and oleylamine (3 ml) in a four neck flask. The mixture of octadecene and oleylamine was previously degassed at 115 °C for at least 1 h. The solution was switched to vacuum to remove the hexanes for 30 min at room temperature and another 10 min at 110 °C. Subsequently, the solution was heated to 280 °C for the shell growth. As soon as the mixture reached 240 °C, shell precursor injection was started using 0.05 M shell precursor solutions in octadecene. Cd(Ol)_2_ (111 μmoles) and TOPSe (111 μmoles) were added over the course of 67 min (injection speed 2 ml h^−1^). InAsCdSe CS QDs were purified using the above described procedure (however using acetone and methanol as non-solvents) and stored in hexanes. In contrast to bare InAs cores, the InAs core-shell QDs were not transferred to a glovebox but stored in air. The size was measured to be 5.1 nm (TEM) and the QDs exhibited a PL peak at 1,296 nm. The resulting InAsCdSe (roughly 86 nmoles, 10% loss through aliquots and purification) were redispersed in octadecene (3 ml) and oleylamine (3 ml), and degassed at room temperature for 30 min and at 100 °C for 10 min. To that solution 3 ml of a 0.05 M Cd(Ol)_2_ and 3 ml of a 0.045 M sulfur solution in octadecene were added (150 μmoles Cd and 135 μmoles S) at 240 °C within 1 h. The final QD diameter was determined to be 6.9 nm (TEM) with a PL emission at 1,307 nm.

### QD phase transfer using PEG-phospholipid micelles

QDs were transferred into aqueous solution by adapting a previously reported procedure^44^. Briefly, QDs (1–2 mg) were mixed with 18:1 PEG2000 PE (1,2-dioleoyl-sn-glycero-3-phosphoethanolamine-N-[methoxy(polyethylene glycol)-2000]) (ammonium salt) (25-50 mg, 1:25 ratio, Avanti Polar Lipids; Cat. No 880130) in chloroform. The mixture was sonicated for 15 min and the solvent was removed under nitrogen flow. All residual solvent was removed by drying the sample under vacuum for 15 min. The dry sample was then redispersed in saline solution to obtain a 1 mg ml^−1^ solution. To assure complete dispersion of particles, the sample was sonicated for 15 min, before it was filtered through a 200 nm syringe filter (Life Sciences, Aerodisc, 0.2 μm HT Tuffryn).

### Mouse imaging

In our custom imaging setup, a 10 W 808 nm laser (Opto Engine; MLL-N-808) is coupled in a 910 μm-core metal-cladded multimode fiber (Thorlabs; MHP910L02). The output from the fiber is passed through a ground-glass plate (Thorlabs; DG10-220-MD) directed over the working area. We used a 1” silver elliptical mirror (PFE10-P01) to direct the emitted light to the InGaAs camera (Princeton Instrument, NIRvana) equipped with doublet lenses (Thorlabs AC254-100-C and AC254-75-C). In between the lenses we employed various 50 nm bandpass filters (Edmund Optics) for the wavelength selective imaging. The laser light was blocked with a doubled coloured glass 2' 850 nm longpass filter.

QD samples emitting at 970, 1,110 and 1,300 nm were transferred to water as described above. 800 μl of the QD_970_ solution, 500 μl of the QD_1,110_ solution and 580 μl of the QD_1,300_ solution were mixed to obtain broad band emission at 808 nm excitation over the sensitivity range of the InGaAs camera. A C57BL/6J mouse (34 g, male, 22 weeks, Jackson Lab) was shaved, anaesthetized and placed under the imaging setup. The mouse was irradiated with 808 nm laser light at an intensity of 60 mW cm^−2^ and 300 μg of QDs dissolved in water were injected via the tail vein. The brain vasculature was imaged using different bandpass filters. To account for the chromatic aberration of the optical system, the camera was refocused to maximize the resolution of the central brain feature after each change of filter. All pictures were background corrected and the emission profile of the fine brain vasculature was analysed using ImageJ.

Animal experiments were conducted in accordance with approved institutional protocols of the MIT Committee on Animal Care. In more than 40 mice injected with different formulations of our SWIR QDs we did not observe any acute toxicity effects of the QDs across many different experiments.

### Absorption spectroscopy

Absorption spectra were recorded on a Cary 5000 UV-Vis-NIR infrared spectrometer from Varian or on an 8453 UV-Vis spectrometer from Agilent and corrected for absorption through solvent and surfactants (octadecene, oleylamine). A small, vertical bump in absorbance at 800 nm that occurs from a detector switchover in the in instrument was corrected for by subtraction of the offset value for wavelengths smaller than 800 nm.

### PL spectroscopy

All samples were excited with a 532 nm diode laser, and emission was collected using a pair of gold-coated off-axis parabolic mirrors, and directed to a single-grating spectrometer (Acton; Spectra Pro 300i). Samples emitting between 700 and 1,100 nm were measured using a on a thinned InGaAs point detector (Thorlabs, DET10N). Samples emitting past 1,400 nm were measured using a liquid nitrogen cooled InAs point detector (Hamamatsu, P7163). Samples with emission peaks between 1,000 and 1,400 nm were measured using a liquid nitrogen cooled InGaAs line camera (Princeton Instruments; OMA V, 512 × 1 pixels).

### PL quantum yield

QY measurements were obtained using an integrating sphere (Labsphere RTC-060-SF). The sample was illuminated using a 785 nm diode laser with an excitation power of 25 mW that was chopped at 210 Hz. The output was collected using a calibrated germanium detector (Newport: 818-IR) through a Stanford Research Systems lock-in amplifying system. A coloured glass longpass filter (Schott Glass RG800 or RG850) was used to block the excitation beam. The sample was placed in a polytetrafluoroethylene (PTFE) capped quartz cuvette and a solvent blank was used to ensure a consistent environment inside the integrating sphere. The measured photocurrent was adjusted to account for the external quantum efficiency of the detector when calculating the QY. Finally, the measured QY was corrected to account for leakage of the excitation light and the transmittance of the filter.

### PL lifetimes

To measure PL dynamics by time-correlated single-photon counting (TCSPC), samples (solutions in glass vials) were excited by a train of 532 nm, ∼80 ps-duration pulses at a repetition rate of 500 kHz generated by a laser diode (PicoQuant; LDH-P-FA-530B). The 2 μs waiting time between pulses ensured that the delayed fluorescence decayed below the noise floor of the detector (<10^−3^ of the peak signal under these conditions). The pump was attenuated to yield 10–100 nW of average excitation power, to ensure a stop rate of ∼5%, and focused to a ∼200 μm diameter excitation spot at the sample. Under these low-fluence excitation conditions, the PL decay dynamics are independent of excitation intensity. The emission from the NCs was collected and imaged onto an InGaAs/InP single-photon counting avalanche photodiode (Micro Photon Devices; $IR-DH-025-C), fitted with a long-pass filter (Chroma Technology Corp.; EP900LP) to suppress the scattered photons from the visible pump. The detector was operated asynchronously from the laser source, with a 2 MHz gate frequency and a 90% duty cycle. Using a PicoQuant PicoHarp and standard software, a decay trace histogram was generated by correlating the times of detection events with the proximal trigger from the pump laser. The time resolution/instrument response, judged from the onset of the response to the impulsive excitation, was ∼300 ps. Accordingly, the measured data are presented without deconvolution, as their most rapid dynamics are more than an order of magnitude slower than the instrument response.

### TEM images

All TEM images were acquired on a JOEL 2010 Advanced High Performance TEM operating at 200 KV with a lanthanum hexaboride cathode. TEM samples were prepared by drop casting a purified solution of QDs from hexanes onto a 400 mesh copper grid with a carbon film (Ted Pella). ImageJ was employed to generate the volume-weighted size distributions shown in Fig. 2a. The images were Fourier transformed using the built-in ‘Bandpass Filter' option. The threshold was adjusted to match the QD dimension and the average 2D projection area of the NCs in the TEM was measured. TEM images of CSS QDs can be found in Supplementary Fig. 10. Assuming a spherical shape, the crystal volume was obtained via eq. (2):


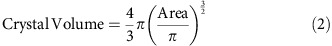


### Elemental analysis

All elemental analysis was performed on a JOEL 2010 Advanced High Performance TEM, operating at 200 KV with a lanthanum hexaboride cathode. The electron beam was spread out to average over 50–100 QDs per measurement and three measurements at different sample locations were performed. Elemental analysis was performed using energy-dispersive X-Ray spectroscopy analysis and INCA software.

### Data availability

The authors declare that the data supporting the findings of this study are available within the article and its Supplementary Information files.

## Additional information

**How to cite this article**: Franke, D. *et al*. Continuous injection synthesis of indium arsenide quantum dots emissive in the short-wavelength infrared. *Nat. Commun.* 7:12749 doi: 10.1038/ncomms12749 (2016).

## Supplementary Material

Supplementary Information Supplementary Figures 1-12, Supplementary Tables 1 & 2, Supplementary Notes 1-3 and Supplementary References.
