# Supplementary material for: Continuous injection synthesis of indium arsenide quantum dots emissive in the short-wavelength infrared
Source: Nat Commun. 2016 Nov 11;7:12749. doi: 10.1038/ncomms12749 (PMC5114595; doi:10.1038/ncomms12749)
Supplement: Supplementary Information — Supplementary Figures 1-12, Supplementary Tables 1 & 2, Supplementary Notes 1-3 and Supplementary References. [file ncomms12749-s1.pdf]

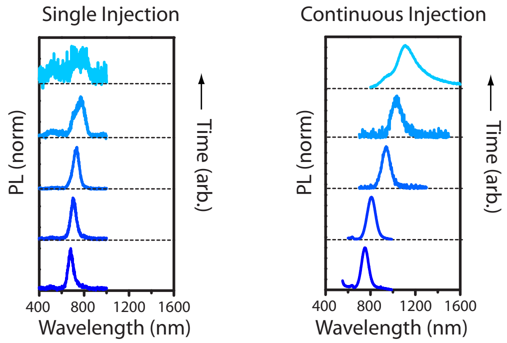

Supplementary Figure 1| Photoluminescence Spectra taken during the Growth of InAs QDs. Left: Single hot injection. **Right:** Hot injection followed by a continuous injection. The respective absorption spectra are displayed in **Fig 1b,e** in the main text.

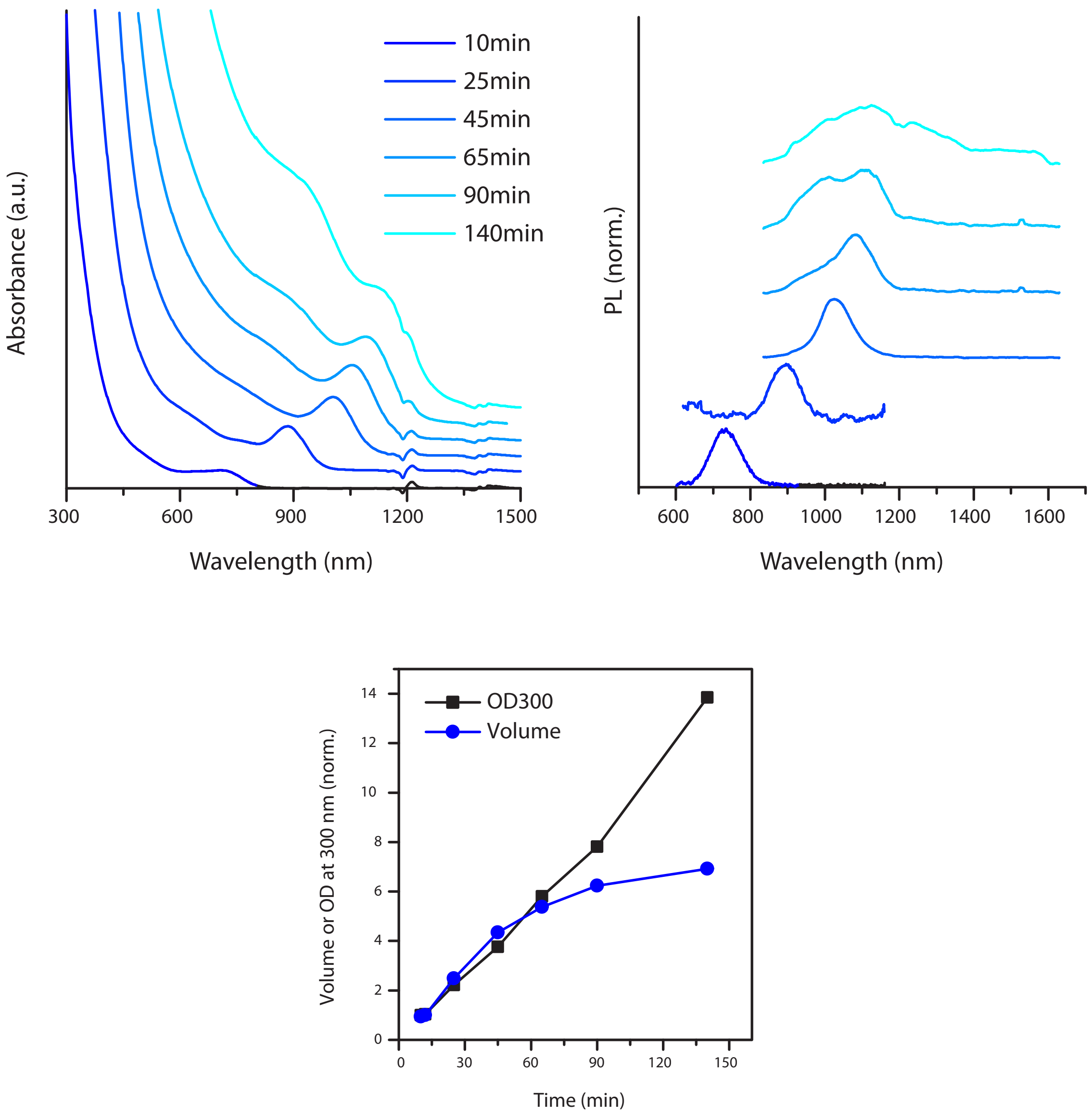

Supplementary Figure 2| Inhomogeneous Linewidth Broadening and Divergence of QD growth. (Top) Absorption and PL spectra taken during the growth of InAs QDs as described in **Fig. 2a** in the main text (injection speed 1 mL h^{-1}). (Bottom) The OD at 300 nm is proportional to the total concentration of InAs in the growth solution. The QD size is calculated based on the absorbance peak using a sizing curve. The curves initially overlap quite well, suggesting that material added to the growth solution is added to existing particles. After about 45 minutes, the particle growth rate appears to slow down and precursor material added to the growth solution does not seem to be exclusively added to existing particles.

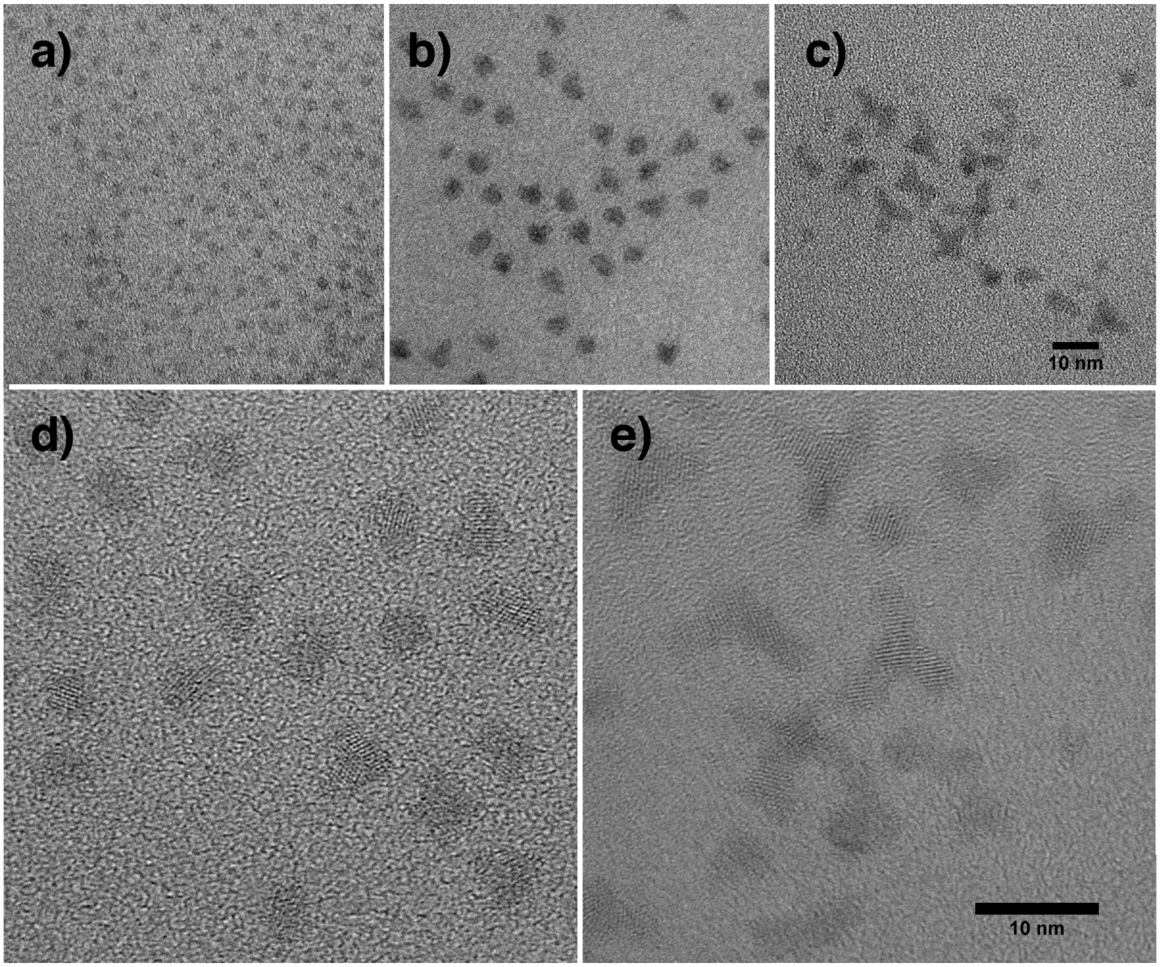

Supplementary Figure 3| Shape Anisotropy of InAs QDs caused by too high Precursor Concentrations during QD Growth. TEM of the (a) 10 minute aliquot, (b) the 45 minute aliquot, and (c) the 140 minute aliquot taken during the growth of InAs QDs as described in **Fig. 2a** in the main text (injection speed 1 mL h^{-1}). (d) and (e) are HRTEM images of the 45 minute and 140 minute aliquots, respectively.

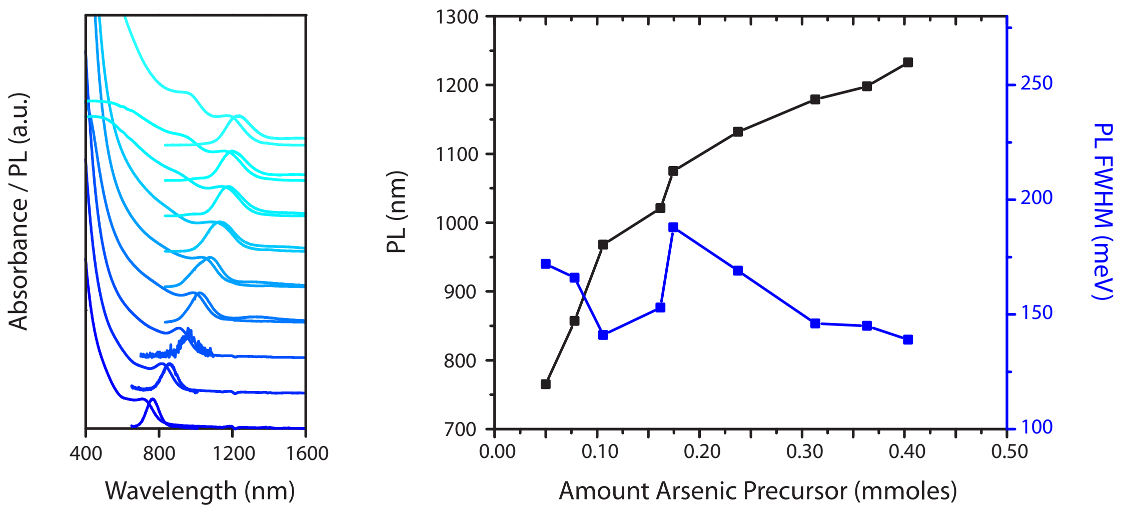

Supplementary Figure 4| Synthesis of InAs QDs from $(iPrDMSi)_3As$. The synthesis of InAs QDs from tris(isopropyldimethylsilyl)arsine ($(iPrDMSi)_3As$) was adapted from the continuous injection synthesis described in the main article. $(iPrDMSi)_3As$ was synthesized as previously described.[1] 1 mmole $In(Acetate)_3$ was added to 4 mmoles oleic acid and 5 mL ODE and the dispersion was degassed at 115°C under vacuum to form $In(Oleate)_3$. The final pressure after 1 hour was measured to be lower than 10 mtorr. The reaction atmosphere was switched to nitrogen and heated to 295°C. Subsequently, 0.05 mmoles of $(iPrDMSi)_3As$ in 1 mL TOP were injected. After 10 minutes a continuous injection of a 0.168 M solution of $(iPrDMSi)_3As$ in ODE was started. The injection speed was set to 2 mL h^{-1} for the first 20 minutes and then switched to 0.15 mL h^{-1} for another 575 min, such that the final amount of arsenic precursor injected into solution equals 0.404 mmoles. The PL peak continuously shifted from 765 nm and a FWHM of 172 meV (10 minutes) to 1233 nm and a FWHM of 139 meV (605 minutes). **Left**: Absorption and PL spectra taken during the growth of InAs QDs from $(iPrDMSi)_3As$. **Right**: Position and FWHM of the PL peak as a function of the amount of arsenic precursor added to the solution.

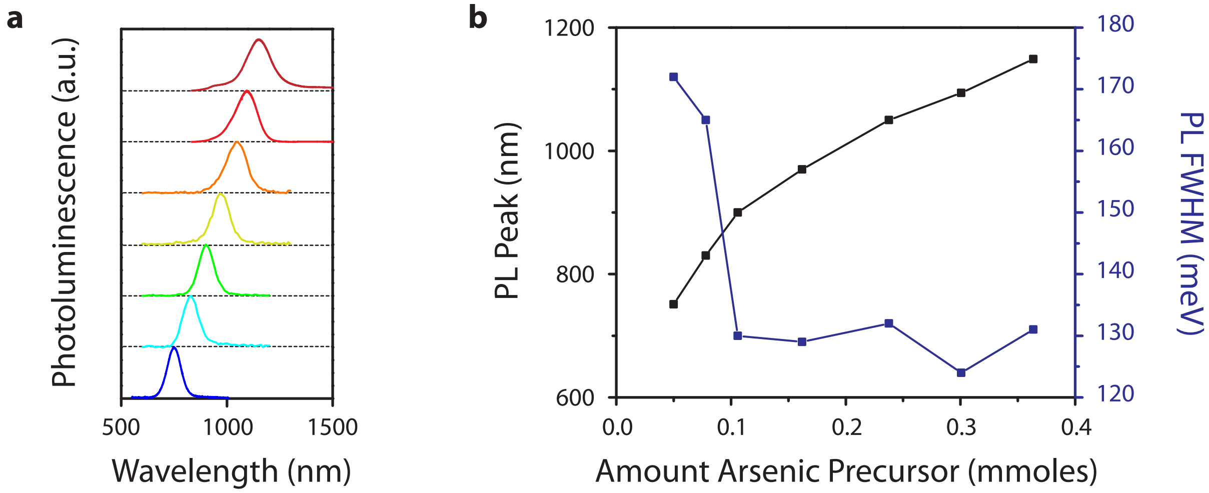

Supplementary Figure 5| Synthesis of InAs QDs from (TMSi)$_3$As. The synthesis of InAs QDs from tris(trimethylsilyl)arsine) ((TMSi)$_3$As) was adapted from the continuous injection synthesis described in the main article. (TMSi)$_3$As was synthesized as previously described.[1] 1 mmole In(Acetate)$_3$ was added to 4 mmoles oleic acid and 5 mL ODE and the dispersion was degassed at 115°C under vacuum to form In(Oleate)$_3$. The final pressure after 1 hour was measured to be lower than 10 mtorr. The reaction atmosphere was switched to nitrogen and heated to 295°C. Subsequently, 0.05 mmoles of (TMSi)$_3$As in 1 mL TOP were injected. After 10 minutes a continuous injection of a 0.168 M solution of (TMSi)$_3$As in ODE was started. The injection speed was set to 2 mL h^{-1} for the first 30 minutes and then switched to 0.15 mL h^{-1} for another 480 min, such that the final amount of arsenic precursor injected into solution equals 0.301 mmoles. The PL peak continuously shifted from 751 nm and a FWHM of 172 meV (10 minutes) to 1149 nm and a FWHM of 131 meV (510 minutes). During the reaction, the formation of a small metallic solid on the side of the flask was noted and the growth solution turned slightly turbid. The resulting aliquots after the injection of 0.238 mmoles arsenic precursor were thus filtered (200 nm syringe filter) before the PL spectrum was measured. We attribute the formation of precipitate in the solution to a decomposition reaction of (TMSi)$_3$As at these elevated temperatures. **Left**: PL spectra acquired during the growth of InAs QDs from (TMSi)$_3$As. **Right**: Position and FWHM of the PL peak as a function of the amount of arsenic precursor added to the solution.

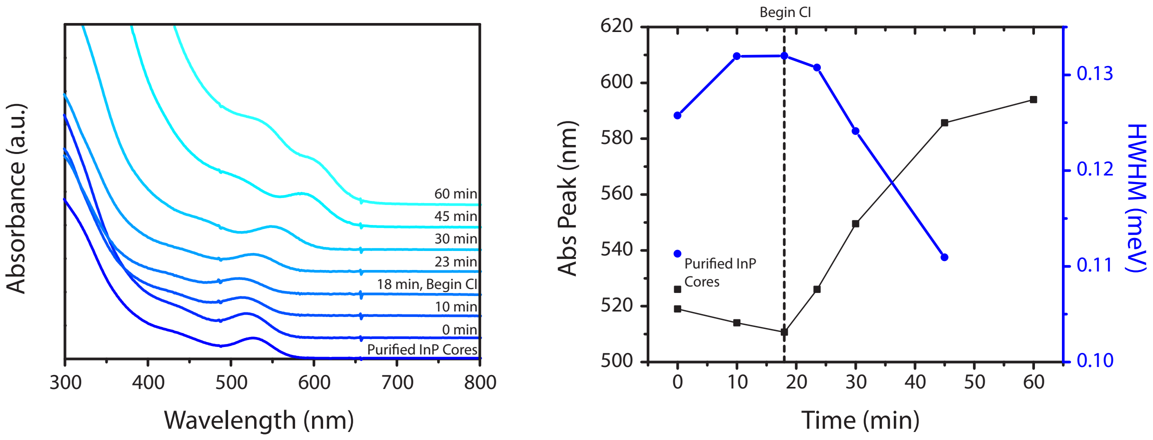

Supplementary Figure 6| Continuous Injection Synthesis of InP QDs. Left: Absorption spectra of aliquots taken during the growth of InP QDs using a continuous injection approach. Right: Upon begin of the injection of precursors, growth sets in (the absorption peak redshifts) which is accompanied by a size focusing (narrowing of the half-width at half maximum, HWHM, of the absorption peak).

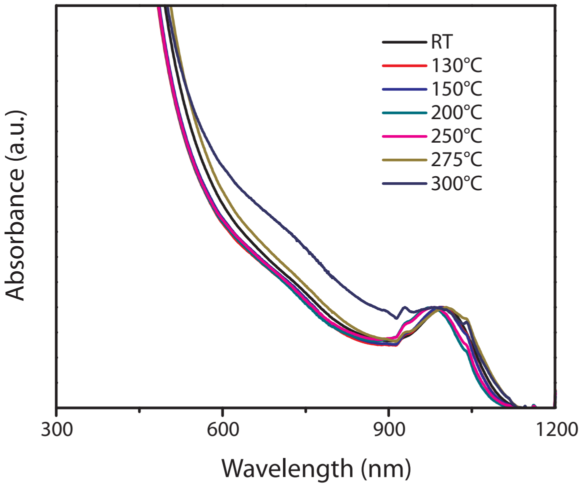

Supplementary Figure 7| Stability of InAs Cores. InAs cores with a diameter of 4.2 nm (measured by TEM) and an absorption maximum at 1008 nm were dispersed in 1:1 mixture of ODE and oleylamine and the solution was degassed at 110°C for 30 minutes. The solution was slowly heated up to 300°C and aliquots were taking at different temperature steps. At temperatures over 275°C a significant broadening of the first excitonic feature was observed, indicating a broadening of QD size distribution and a decrease in colloidal stability. Absorption spectra were normalized to the first excitonic feature.

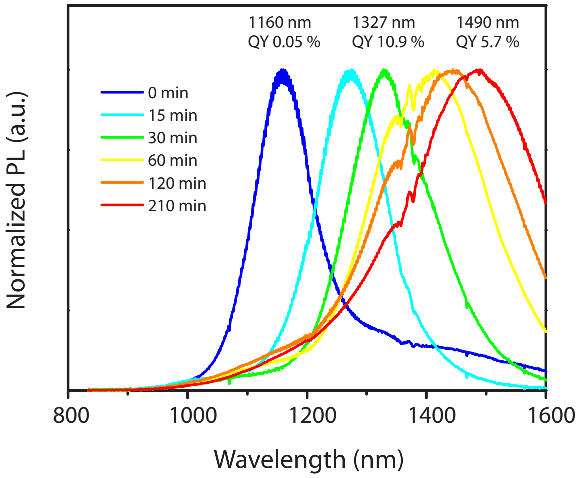

Supplementary Figure 8| Shift of PL Peak Position upon Growth of CdSe onto InAs QDs. InAs cores emitting at 1160 nm were dispersed in a 1:1 mixture of ODE and oleylamine and overcoated with a 0.05 M solution of $Cd(Ol)_2$ in ODE and a 0.05 M solution of TOPSe in ODE. The emission peak of the InAs cores is redshifted by more than 300 nm. The QY passes through a maximum of 10.9 % at an emission wavelength of 1327 nm and then drops back to 5.7 % for the final sample, emitting at 1490 nm.

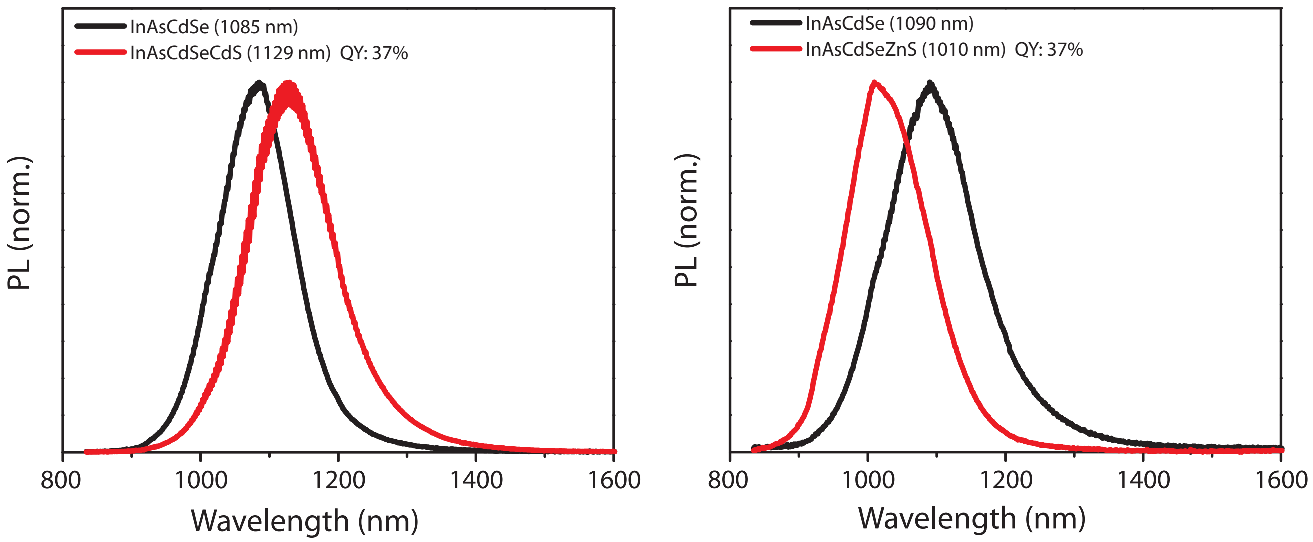

Supplementary Figure 9| Comparison of CdS and ZnS as respective Outer Shell Materials. Two samples of InAsCdSe core-shell QDs emitting at 1085 nm and 1090 nm were overcoated with CdS and ZnS, respectively. Overcoating with ZnS causes a blueshift of around 80 nm, while overcoating with CdS causes a redshift of around 44 nm. Both methods yield core-shell-shell particles with identical QYs.

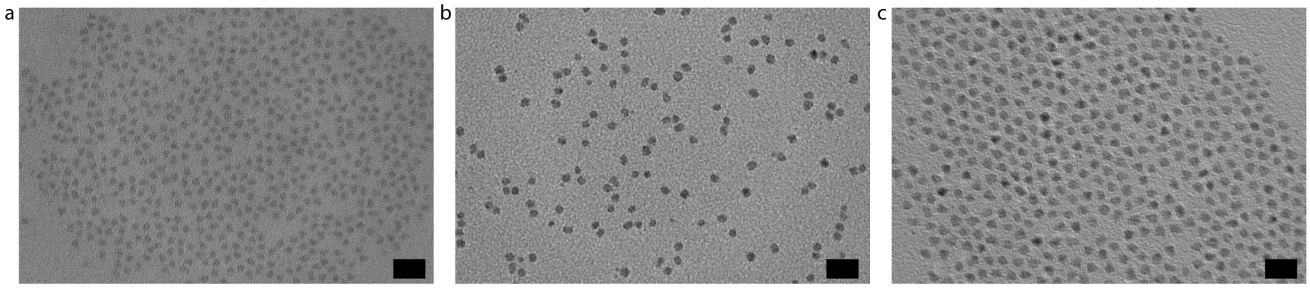

Supplementary Figure 10| TEM of InAsCdSeCdS CSS QDs. TEM images of **a** InAs cores, **b** InAsCdSe and **c** InAsCdSeCdS with emission peaks at 1039 nm, 1296 nm and 1290 nm, respectively. Scale bar equals 20 nm in all images.

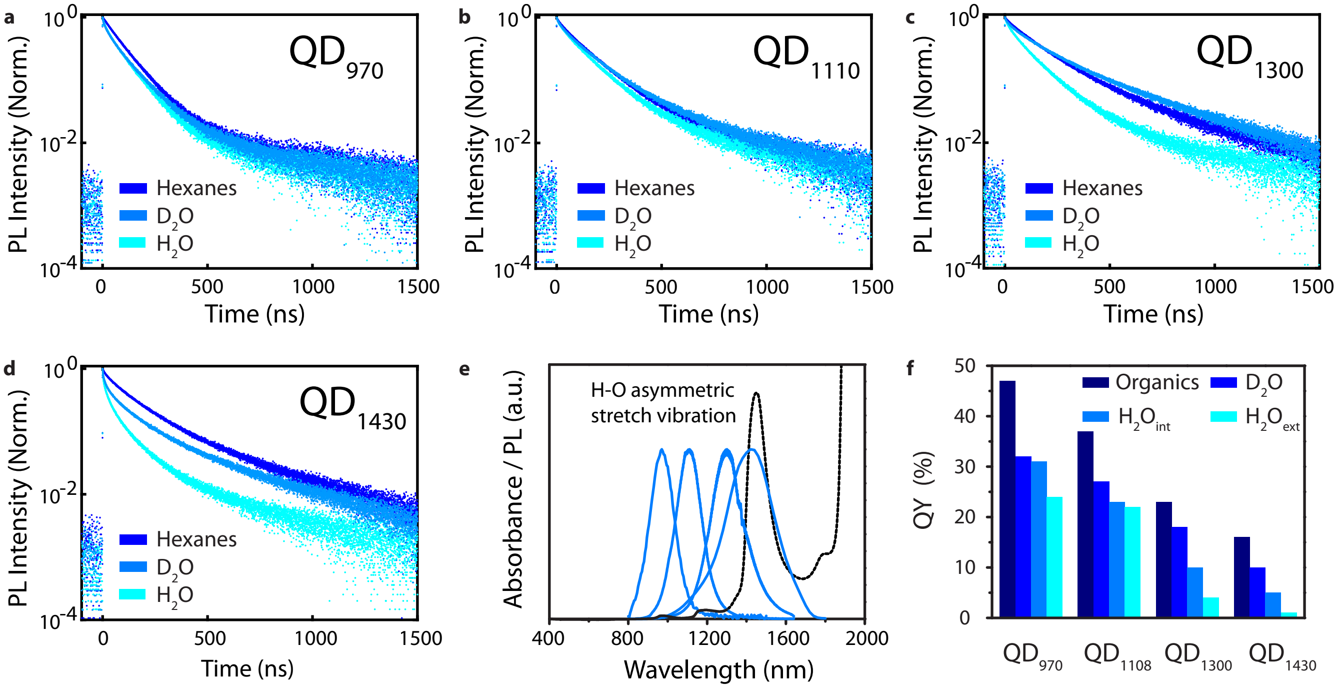

Supplementary Figure 11| Determination of the intrinsic QY of CSS QDs in H2O. a-d PL lifetime curves of four QD samples emitting in the sensitivity range of InGaAs cameras. Samples **a** and **d** are comprised of **a** InAsCdSeZnS CSS structure, while **b** and **c** exhibit an InAsCdSeCdS structure. All PL lifetime curves were recorded in Hexanes, H_2O and D_2O with 532 nm excitation at 500 kHz, 32 ps intrinsic resolution (re-binned 8-fold, background subtracted and normalized). While QD_{970} and QD_{1110} show almost identical dynamics in all three solvents, QD_{1300} and QD_{1430} exhibit a much faster decay of PL intensity for the samples dispersed in water. **e** Overlay of PL spectra of the four QD samples in organic solution and the absorption spectrum of H_2O. **f** QY of all four samples in organic solutions, H_2O (extrinsic) and D_2O and a calculated value for the intrinsic QY in H_2O. QYs were measured in a 1x1 cm^2 cuvette with a concentration of 1 mg QDs mL^{-1} solvent.

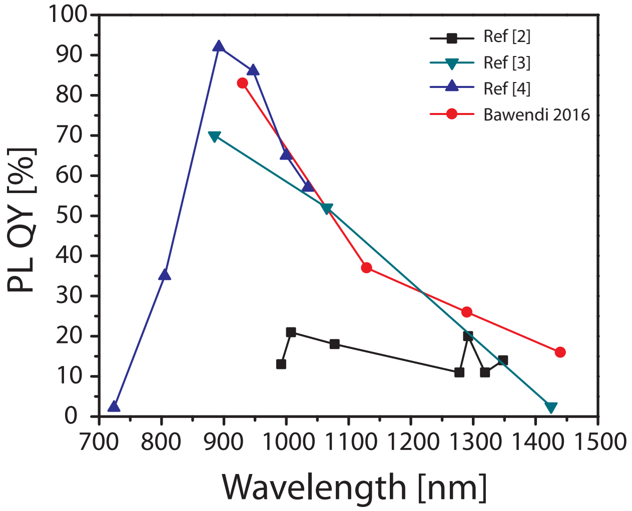

Supplementary Figure 12| Comparison of CSS QDs to other InAs-based QDs reported in literature.[2–4]

Supplementary Table 1| Elemental Analysis of InAsCdSeCdS CSS QDs. InAs QDs (Diameter: 4.7 nm (from sizing curve), PL peak at 1039 nm, 96 nmoles QDs) were overcoated with 112 µmoles of $Cd(Oleate)_2$ and TOPSe to obtain InAsCdSe core-shell QDs (PL peak at 1296 nm). After purification InAsCdSe QDs (86 nmoles) were overcoated with 150 µmoles $Cd(Oleate)_2$ and 135 µmoles ODE-S to obtain InAsCdSeCdS CSS QDs (PL peak at 1307 nm).

Elements	Experimental Atomic Ratio (EDX)	Theoretical Ratio
In : As	1.0 : 1	1.0 : 1
Cd : (Se+S)	1.2 : 1	1.1 : 1
S : Se	1.9 : 1	1.4 : 1
Cd : In	7.0 : 1	3.0 : 1

Supplementary Table 2| Elemental Analysis of InAsCdSeZnS CSS QDs. InAs QDs (Diameter: 3.9 nm (from sizing curve), PL peak at 901 nm, 72 nmoles QDs) were overcoated with 50 µmoles of $Cd(Oleate)_2$ and TOPSe to obtain InAsCdSe core-shell QDs (PL peak at 1090 nm). After purification InAsCdSe QDs (32 nmoles) were overcoated with 38 µmoles $Zn(Oleate)_2$ and 34 µmoles ODE-S to obtain InAsCdSeZnS CSS QDs (PL peak at 1023 nm).

Elements	Experimental Atomic Ratio (EDX)	Theoretical Ratio
In : As	1.6 : 1	1.0 : 1
Cd : Se	1.2 : 1	1.0 : 1
Zn : S	0.5 : 1	1.1 : 1
Zn : Cd	1.1 : 1	1.7 : 1
Cd : In	0.9 : 1	1.3 : 1
Zn : In	0.9 : 1	2.1 : 1
Se: In	0.7 : 1	1.3 : 1
S : In	1.7 : 1	1.9 : 1

Supplementary Note 1| Inhomogeneous Linewidth Broadening through Shape Anisotropy

The results from this paragraph and Supplementary Fig. 2-3 were first discussed and published in the Ph.D. thesis of D.K. Harris in 2014 and were included for completion.[5]

To investigate the slowdown in growth rate after the particles reach sizes of about 4.5-5 nm (absorbance peaks approximately around 1000-1100 nm), we compared the particle growth rate with the rate of material added to the reaction solution. During the CI synthesis described in **Fig. 2a** in the main text (1 mL h^{-1}) aliquots were removed periodically and characterized by absorbance and photoluminescence spectroscopy to monitor the evolution of QD size and size distribution. As described in the main article, avoiding a secondary nucleation step is key to obtaining a narrow size distribution. To measure whether all precursor material added to the solution is incorporated into existing crystals, we used:

- UV absorbance: Absorbance of short wavelength light scales with InAs material in solution and should be independent of particle size.[6,7]
- QD size from sizing curve. To obtain the QD size from the first excitonic feature in the absorption spectrum, the sizing curve of Yu et al. was employed.[8]

Particle size and total InAs concentration are normalized to the aliquot taken immediately before the beginning of the continuous injection and plotted in Supplementary Fig. 2. These data clearly show that particle growth is commensurate with precursor addition initially. During this time, the PL linewidth drops rapidly before becoming very broad after about 65 minutes. The minimum FWHM achieved during this synthesis occurs at about 45 minutes, and corresponds to the point in Supplementary Fig. 2 where the material added and the QD volume diverge.

The most obvious interpretation of this result is that material added after 45 minutes results in the formation of new particles. To investigate the nature of the broadening of the linewidth during growth, TEM was used to characterize the aliquots taken at 10 minutes, 45 minutes, and 140 minutes. Supplementary Fig. 3 shows that the initial 10 minute aliquot and the 45 minute aliquot have approximately spherical shapes with a mean size measured to be 4.5 nm from TEM images. However, the particles in the 140 minute aliquot have asymmetric shapes. These shapes seem to have lobes that do not share the symmetry of the zincblende crystal structure. The average lobe diameter was measured to be approximately 3.5 nm. This is consistent with a growth trajectory in which the particles of 4.5 nm diameter coalesce with smaller particles that are not fully absorbed into the parent to form a spherical shape. Therefore, it appears that the broadening in the optical spectra is driven in part by shape inhomogeneity.

Supplementary Note 2| Continuous Injection Synthesis of InP QDs

The results from this paragraph and Supplementary Fig. 6 were first discussed and published in the Ph.D. thesis of D.K. Harris in 2014 and were included for completion.[5]

Although the focus of our work so far has been on InAs QDs, we have also attempted to make InP QDs using a continuous injection strategy. However, due to the chemistry of InP synthesis, we had to slightly adjust our strategy. In contrast to the growth of InAs QDs,[9] InP QD growth is known to be negatively affected by the presence of excess carboxylic acid,[10,11] but we believe that the presence of some acid is beneficial to the continuous injection process as it may preferentially digest smaller particles. Therefore, we used a two-pot approach, where we synthesized and purified InP QDs with an absorbance peak at 525 nm for use as seeds. We then redispersed these seeds in an In(Myristate)$_3$ solution for continued growth.

For seed growth, 1 mmole of In(Acetate)$_3$ was mixed with 3 mmoles of myristic acid and 5 mL of ODE. This solution was heated under vacuum at 115°C for 60 minutes to remove acetic acid displaced during the formation of In(Myristate)$_3$. The resulting clear solution was heated to 150°C under argon, and 0.5 mmoles of (TMSi)$_3$P dissolved in 1 mL of TOP were injected. The temperature controller was immediately switched to 275 °C for growth. The solution was cooled 7 minutes after injection. 0.5 mL of the growth solution was removed to atmosphere, and the residual particles were purified by adding acetone and centrifuging the turbid solution. Hexane was used to redisperse the pellet of InP QDs at the bottom of the centrifuge tube. This was repeated once more, and the resulting stock solution was diluted for measurement of its absorbance spectrum and found to have an OD of 100 at 300 nm. A solution of 1 mmole of In(Myristate)$_3$ in ODE was prepared by again adding 1 mmole of In(Acetate)$_3$, this time with 3.1 mmoles of myristic acid. Acetic acid was removed by evacuating at 115 °C. The reaction vessel was allowed to cool and 630 mg of the solution of InP cores was added to the reaction vessel and vacuum was applied briefly to remove the hexanes as the reaction solution was heated to 250 °C. The reaction timer was started as the solution reached 150°C. Aliquots were removed at $t = 0$ min, $t = 10$ min, and $t = 18$ min, prior to starting the continuous injection at $t = 18$ min. Absorption spectra were taken of these aliquots, and they reveal that the InP cores were somewhat unstable in the reaction solution. The absorption peak blueshifted from 525 nm to 510 nm at $t = 18$ min and the half width at half max (HWHM) of the absorption peak increased from 111 meV for the initial seeds to 132 meV for the aliquot removed at $t = 18$ min. Supplementary Fig. 6 shows that as soon as the continuous injection is started, we note an onset of QD growth (the absorption maximum redshifts about 80 nm) and a narrowing of size distribution (the HWHM of the absorption maximum narrows by about 20 meV). However, it is apparent that as the reaction proceeds, the particle growth slows and the features become less defined as it did with InAs QDs at faster injection speeds. TEM images suggest that both shape inhomogeneity as well as size inhomogeneity contribute to the spectral broadening observed.

Supplementary Note 3| Determination of intrinsic QYs in Water.

As phase transfer techniques are known to negatively affect QYs for QD systems, for example by altering QD ligand chemistry and surface passivation, QDs typically exhibit lower QYs in aqueous media, compared to organic solvents. However with regards to SWIR emissive QDs, the QY in aqueous media may be further decreased by both near-field interactions, such as non-radiative energy transfer to nearby water molecules and far-field effects such as reabsorption of emitted photons through the solvent. While enhanced near-field interactions, such as FRET, can be considered intrinsic properties of the fluorophore-solvent system, reabsorption processes strongly depend on the measurement setup, sample concentration, cuvette length, or geometry of the integrating sphere, which impedes a comparison across literature.

To extract the intrinsic, setup-independent QY of SWIR QDs in aqueous solution corrected for reabsorption events, we measured PL lifetimes and the extrinsic QYs of four QD samples in water (saline solution, H_2O) and heavy water (D_2O) (see Supplementary Fig. 11 a-e). As D_2O does not exhibit a strong absorption band in the SWIR region, due to the mass difference between hydrogen and deuterium, energy transfer interactions between QDs and D_2O are strongly suppressed. In contrast to transferring QDs from organic solvents to H_2O, the transfer from H_2O to D_2O can easily be carried out over repeated dialysis of the sample with D_2O. Assuming that dialysis does not significantly alter the QD surface and thus the radiative lifetimes of CSS QDs, we calculated the FRET efficiency by comparing the integrated PL lifetimes (biexponential fit between $0<t<500$, equation 1) in both H_2O and D_2O.[12] The ratio of the integrated PL lifetimes in H_2O and D_2O were multiplied with the QY in D_2O to yield the intrinsic QY of the sample in H_2O (equation 2), including all near-field interactions but not the setup and concentration dependent reabsorption effect.

$$C_i = \int_0^\infty A_1 e^{-\frac{t}{\tau_1}} + A_2 e^{-\frac{t}{\tau_2}} \quad (1)$$

$$QY(H_2O_{int}) = QY(D_2O) * \frac{C_{H_2O}}{C_{D_2O}} \quad (2)$$

To exchange H_2O for D_2O, 1 mL of a 1 mg mL^{-1} QD solution in H_2O was transferred to a centrifugal filter (Amicon Ultra, Ultracel 30K MWCO) and centrifuged for 8 min at 7 krpm so that almost all H_2O was removed. 1 mL of D_2O was added and the sample was centrifuged for another 8 minutes at 7 krpm. This procedure was repeated 3 times. Afterwards the sample was redispersed in 1 mL of D_2O.

Supplementary Fig. 11 f shows the measured QYs for all four samples in organic solutions (hexanes, CCl4, or trifluoro trichloroethane), D_2O, H_2O as well as the calculated intrinsic QY in H_2O. It is evident that especially the two samples emitting close to the water band, QD_{1300} and QD_{1430}, show a strong difference in fluorescence lifetimes in H_2O and D_2O (Supplementary Fig. 11 c,d). Comparing the QY in D_2O with the calculated, intrinsic QY in H_2O we find a relative QY reduction due the near-field interaction between QDs and H_2O of 3%, 15%, 44% and 50% for samples emitting at 970 nm, 1110 nm, 1300 nm, and 1430 nm, respectively. The increase in non-radiative energy transfer with increasing emission wavelength is in good qualitative agreement with the increase in overlap between QD emission and the water absorption band at 1450 nm (Supplementary Fig. 11 e).

Supplementary References

[1] Franke, D., Harris, D. K., Xie, L., Jensen, K. F. & Bawendi, M. G. The unexpected influence of precursor conversion rate in the synthesis of III-V quantum dots. *Angew. Chemie Int. Ed.* **54**, 14299–14303 (2015).

[2] YunWei & Banin, U. Growth and properties of semiconductor core/shell nanocrystals with InAs cores. *J. Am. Chem. Soc.* **122**, 9692–9702 (2000).

[3] Aharoni, A., Mokari, T., Popov, I. & Banin, U. Synthesis of InAs/CdSe/ZnSe core/shell1/shell2 structures with bright and stable near-infrared fluorescence. *J. Am. Chem. Soc.* **128**, 257–264 (2006).

[4] Xie, R. & Peng, X. Synthetic scheme for high-quality InAs nanocrystals based on self-focusing and one-pot synthesis of InAs-based core-shell nanocrystals. *Angew. Chemie* **120**, 7791–7794 (2008).

[5] Harris, D. K. Synthesis and Characterization of Infrared Quantum Dots. (Massachusetts Institute Of Technology, 2014).

[6] Harris, D. K. & Bawendi, M. G. Improved precursor chemistry for the synthesis of III-V quantum dots. *J. Am. Chem. Soc.* **134**, 20211–20213 (2012).

[7] Leatherdale, C. A., Woo, W.-K., Mikulec, F. V. & Bawendi, M. G. On the absorption cross section of CdSe nanocrystal quantum dots. *J. Phys. Chem. B* **106**, 7619–7622 (2002).

[8] Yu, P. et al. Absorption cross-section and related optical properties of colloidal InAs quantum dots. *J. Phys. Chem. B* **109**, 7084–7087 (2005).

[9] Baek, J. Microchemical Systems for the Synthesis of Nanostructures: Quantum Dots. (Massachusetts Institute Of Technology, 2012).

[10] Baek, J., Allen, P. M., Bawendi, M. G. & Jensen, K. F. Investigation of indium phosphide nanocrystal synthesis using a high-temperature and high-pressure continuous flow microreactor. *Angew. Chemie Int. Ed.* **50**, 627–630 (2011).

[11] Tamang, S., Lincheneau, C., Hermans, Y., Jeong, S. & Reiss, P. Chemistry of InP Nanocrystal Syntheses. *Chem. Mater.* **28**, 2491–2506 (2016).

[12] Clegg, R. M. in *FRET and FLIM Techniques. Laboratory Techniques in Biochemistry and Molecular Biology* 1–57 (Elsevier, 2009).
